# Secondary Prevention in Patients with Coronary Heart Diseases: What Factors Are Associated with Health Status in Usual Primary Care?

**DOI:** 10.1371/journal.pone.0051726

**Published:** 2012-12-26

**Authors:** Dominik Ose, Justine Rochon, Stephen M. Campbell, Michel Wensing, Jan van Lieshout, Lorenz Uhlmann, Tobias Freund, Joachim Szecsenyi, Sabine Ludt

**Affiliations:** 1 University Hospital of Heidelberg, Department of General Practice and Health Services, Heidelberg, Germany; 2 Radboud University Nijmegen Medical Centre, Scientific Institute for Quality of Healthcare, HB Nijmegen, The Netherlands; 3 University of Heidelberg, Institute of Medical Biometry and Informatics, Heidelberg, Germany; 4 Health Sciences – Primary Care Group, University of Manchester, Manchester, United Kingdom; S.G.Battista Hospital, Italy

## Abstract

**Background:**

For patients with coronary heart diseases a substantial part of secondary prevention is delivered in primary care. Along with the growing importance of prevention, health-related quality of life (HRQoL) is an indicator of patient-centered care that has gained increased attention. Different approaches for reorganization in primary care have been associated with improvements in HRQoL. However, these are often results of complex interventions. Evidence on aspects concerning usual primary care that actually have an impact on HRQoL remains scarce. Therefore, this observational study aimed to identify factors which are associated with HRQoL in usual primary care at practice and patient-level.

**Methods:**

This observational study was conducted in eight European countries. We were able to match data from survey instruments for 3505 patients with coronary heart disease (CHD) in 228 practices. A multilevel analysis was performed to identify associations of EQ-5D scores at patient and practice-level.

**Results:**

After dropping patients with missing information, our cohort consisted of 2656 patients. In this sample, 30.5% were female and the mean age was 67.5 years (SD 10.1). The final model included a total set of 14 potential explanatory variables. At practice-level no variable was associated with EQ-5D. At patient-level, lower education (r = −0.0381, p<0.0001), female gender (r = −0.0543, p<0.0001) and a higher number of other conditions (r = −0.0340, p<0.0001), had a strong negative effect on HRQoL. Strong positive associations with HRQoL were found for a good medication adherence (Morisky) (r = 0.0195, p<0.0001) and more positive evaluations of physicians' clinical behavior (r = 0.0282, p = 0.002). In terms of HRQoL no differences between single-handed and group practices exist.

**Conclusion:**

The results of our study suggest that a better patient-physician relationship rather than organization of CHD care is associated with higher HRQOL in the primary care setting. The results may imply that interventions to improve HRQoL require a strong patient-centered approach.

## Introduction

Cardiovascular disease (CVD), and in particular coronary heart disease (CHD), are major causes of morbidity and premature death and make a substantial contribution to escalating health care costs in developed countries [1], [2]. Accordingly, the treatment and prevention of CVD is a priority for health care systems; especially the modification of risk factors, e.g. hypertension, high cholesterol levels or cigarette smoking, by means of a healthy lifestyle or medication is essential [3]. These and other activities helped to reduce the death rate due to heart diseases in recent decades [4].

However, despite the availability of statins and other pharmacologic agents, e.g. aspirin or beta-blockers, the rate of improvement has slowed down or stopped [5]. To address the “unmet potential for cardiovascular disease prevention” [6], various initiatives have been tried. A common remit of these endeavors, like the “2020 Impact Goals” [7] of the American Heart Association, the “Million Hearts” [8] initiative or the development of quality indicators for CVD prevention in Europe [9], is to focus more on prevention and health promotion, rather than solely on treating diagnosed disease. Such an approach requires a more intensive cross-linking between health care and community based interventions.

Primary care, as a bridge between personal health care and community health care plays a crucial role in this context. A substantial part of prevention and chronic care for CHD is delivered in this sector [10], [11]. However, concepts such as the Chronic Care Model [12], Guided Care [13], GRACE [14] (Geriatric Resources for Assessment and Care for Elders) or PACE [15] (Program of All-inclusive Care for Elderly), offer frameworks for the advancement of primary care that advocate a stronger integrated community focus.

Implementing such approaches, which have been shown to improve health care in numerous clinical studies [16]–[18], requires a strong patient's perspective and involvement. From the patient's perspective not merely the disease, but rather the impact of disease and treatment on daily life is important [19]. In this context “health status” characterizes the range of manifestation of diseases in a given patient, including symptoms and functional limitations. The discrepancy between actual and desired functional capacity is described as health related quality of life (HRQoL). Particularly for patients with chronic conditions, this perspective has a special meaning [20].

Along with the growing importance of prevention, HRQoL as an outcome has gained increased attention in the last years. Various studies have shown the negative impact of disease-specific conditions (e.g. heart failure, hypertension, comorbidities) and sociodemographic factors (e.g. sex, income, and ethnicity) on HRQoL for CHD patients [21], [22]. On the other hand, new concepts have been associated with improvements in HRQoL [23], [24].

However, these are often study results of complex interventions. Evidence on aspects concerning usual primary care that actually have an impact on HRQoL remains scarce. Especially predictors and determinants of optimum HRQoL as a guide for further development, to date are missing [22]. To fill this void, this observational study aimed to identify factors which are associated with HRQoL in usual primary care at practice and patient-level in patients with CHD. With awareness of the ongoing discussion about the future of small and single-handed practices [25], [26], single and group practices were considered separately.

## Methods

### Study Sample

**Figure 1:**
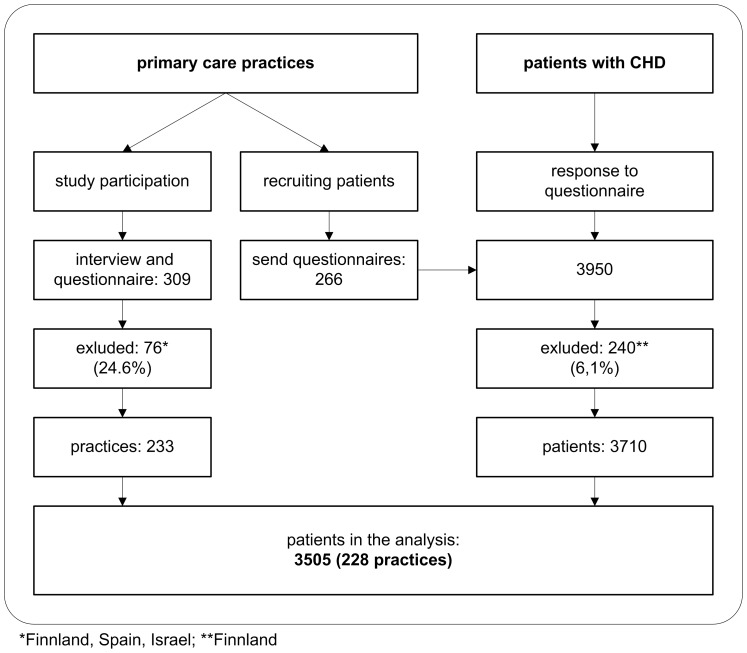
Data flowchart.

This analysis was conducted as part of the European Practice Assessment (EPA) - Cardio project (2006–2009). To improve cardiovascular health care in Europe, in the first stage of the project (2006–2007) instruments and methods for assessing cardiovascular risk management and prevention in primary care were developed and tested [9]. In a second stage of the EPA-Cardio project (2008–2009), a cross-sectional observational study using the EPA-Cardio instrument was conducted. A comprehensive sample of countries in North, West, South and Central Europe participated in this study (i.e. Austria, Belgium, England, Finland, France, Germany, Netherlands, Slovenia, Spain, and Switzerland) [27]. In this part of the study Spain was excluded because only data from medical records were collected and Finland due to insufficient data quality. Israel was only involved in the practice survey (Figure 1). Ethics committees of all participating countries approved the study.

For this study, general practices were recruited by the national research teams with an aim of including 36 practices in each country. Practices were randomly chosen according to the national distribution of general practices across the countries with the intention of achieving a representative country sample. In each practice, 30 patients with CHD were randomly sampled (assuming 50% response) and invited to participate. Inclusion and exclusion criteria are listed in Table 1. All general practitioners (GPs) and all patients provided their written informed consent to participate in this study.

**Table 1:** Inclusion and exclusion criteria for patients with CHD.

Inclusion criteria	Exclusion criteria
Documented diagnosis, ICD 10 code: I20-I25, ICPC-2 code: K74-76	Terminal illness, cognitive disorders (e.g. dementia), psychiatric diseases (e.g. schizophrenia) and lack of language knowledge

This article is one of two analyzing HRQoL in primary care. Within the EPA-Cardio project HRQoL was considered for patients at risk for CVD and for patients with diagnosed CHD. The complete sample comprised over 7,000 cases. Because ‘patients at risk for CVD’ and ‘patients with diagnosed CHD’ are different patient groups with different care needs and treatment plans, we decided to publish the results for both groups separately. The results for “patients at risk” were published elsewhere [28].

### Study Measures

The data collection was based on the EPA-Cardio instrument. At practice-level, team members completed a questionnaire and the leading GP was interviewed using a standardized guide. These instruments contained questions to characterize the practice according to size, location or number and function of practice staff. Quality indicators (QI) that were developed during the EPA-Cardio project [9] and derived from the EPA practice-management instrument [29] were converted into questions for the practice team.

At patient-level, participants received a questionnaire including items about patient characteristics (e.g. age, gender, education and marital status) and care delivery (e.g. practice attendance, referral to exercise program). Additionally, a questionnaire on medication adherence [30], the EQ-5D and a patient satisfaction instrument (EUROPEP) were included.

The EUROPEP (European Project on Patient Evaluation of General Practice Care) instrument is a multidimensional instrument comprising 23 questions on evaluation of specific aspects of general practice care, using a five-point answering scale. Within the two dimensions ‘clinical behavior’ (16 questions) and ‘organization of care’ (7 questions), the patients were asked questions about the clinical behavior of their general practitioner like ‘Making it easy for you to tell him or her about your problems’ or the organization of care like “Getting an appointment to suit you” [27].

The EQ-5D [31], [32], which was used as the main outcome, is a generic instrument for describing and valuing health, and is available in more than 50 languages. The EQ-5D defines health in terms of five dimensions: mobility, self-care, usual activities, pain or discomfort, and anxiety or depression. Each dimension is divided into three levels, indicating no problem, some or moderate problems or extreme problems. The EQ-5D score (indicating HRQoL) ranges from 0 to 1 and can be calculated by applying scores from the EQ-5D preference weights elicited from the general population. The maximum score of 1 indicates the best health state.

For this study, the EQ-5D score was calculated using the value set for the European population. The published evidence supports the validity and reliability of the EQ-5D as an outcome measure within cardiovascular diseases [33]. The minimal importance difference (MID) in health status of patients with CHD is settled within 0.03 points on the EQ-5D score [34].

### Data Analysis

The main study outcome was HRQoL as measured by the EQ-5D score. This study tested a total set of 14 potential explanatory variables as predictors for HRQoL (see Table 2): 12 variables at patient-level and 2 variables on practice-level. To describe practice characteristics, as explanatory variables, we aggregated the items of the practice questionnaire and interview using the homogeneity analysis by alternating least squares (HOMALS). With this analysis, we identified 32 binary items with discrimination measures over 0.4 in two dimensions ‘practice quality management’ (15 items) and ‘practice CVD care’ (17 items) (Table 3). Scores were calculated by summing up the number of ‘yes’- answers resulting in a score range from zero to 15 for the practice quality-management score and from 0–17 for the CVD-care score respectively. Additionally, we grouped practices according to the full-time equivalent (FTE) of GPs in single-handed practices (FTE≤1) and group practices (FTE>1) to appraise differences in a subgroup analysis.

**Table 2:** Explanatory variables included in the multilevel analysis.

Variables	Categories/Scoring
**Practice-level**	
Quality-management score	Continuous: sum score of ‘yes-answers’; range: 0–15
CVD-care score	Continuous: sum score of ‘yes-answers’; range: 0–17
**Patient-level**	
*Patient characteristics*	
Gender	2 categories: female; male
Age	Continuous: age divided by 5
Marital status	2 categories: married/cohabitating; single/separated/divorced/widowed
Education	Years in school; 2 categories : ≤9 years; >9 years
Number of other conditions	Continuous: sum score (range: 0–11) of all patient reported symptoms or conditions including ‘high blood pressure’, ‘high cholesterol’, ‘diabetes’, ‘angina’, ‘history of heart attack’, ‘history of PCI or bypass’, ‘symptoms of heart failure’, ‘transient ischemic attacks’, ‘history of stroke’, ‘peripheral artery disease’ or ‘symptoms of depression’
Body Mass Index	2 categories: up to 30; more than 30
*Care delivery*	
Being patient in practice	3 categories: up to 2 years; 3 to 7 years; more than 7 years
Practice attendance last year	3 categories: up to 3 times/year; 4 to 7 times/year; more than 7 times/year
Referral to exercise program	2 categories: Yes; No/don't Know
Medication adherence (Morisky)	Continuous sum score (4 items): 0–4 (best)
Patient satisfaction (EUROPEP)	
Clinical behavior	Continuous: (EUROPEP dimension ‘clinical behavior’ 16 items) mean: 1–5 (best)
Organization of care	Continuous: (EUROPEP dimension ‘organization of care’ 7 items) mean: 1–5 (best):

**Table 3:** Practice characteristics.

Quality management (15 items)
• Does the practice use a computer-supported patient file system?
• Is the computer used for creating medication prescriptions?
• Does the practice have a procedure for the management of patient information in relation to detailed examination results and the documentation of measures that were taken (e.g., blood examinations)?
• Does the practice have a procedure for the management of patient information in relation to the review of detailed examination results by the doctor (in terms of outgoing needs)?
• Do the practice doctors have direct access to medical guidelines (either on paper or electronic) in their treatment rooms?
• In general: Is practice staff allowed to contact or recall patients?
• Does the practice produce a quality report?
• Has the practice undertaken at least one clinical audit in the last 12 months?
• Did you set standards regarding this clinical audit (defined the target)?
• Did you collect data regarding this clinical audit?
• Did you evaluate the result?
• Were you able to improve the quality regarding this clinical audit topic?
• Does the practice have a critical incident register?
• Did the practice have a team meeting about quality improvement relating to CVD at least once in the last 15 months?
• Did the practice participate in cardiovascular quality improvement projects?

CVD care (17 items)
• Does the practice use case finding methods to detect patients with cardiovascular risk factors?
• Does the practice use a system for recalling patients with cardio vascular diseases?
• Does the practice use a system for recalling patients with diabetes?
• Does the practice use a system for recalling patients with hypertension?
• Does the practice use a system for recalling populations at risk for preventive care regarding cardio vascular diseases?
• Does the practice use a system for recalling populations at risk for preventive care regarding influenza?
• Does the practice have a procedure for smoking cessation (e.g. with the Minimal Intervention Strategy)?
• Does the practice participate in public health care programmes on life style (physical exercise, stop smoking)?
• Did all nurses attend ≥ one training/continuing medical education event on CVD within the last 5 years?
• Did nurses take part in local/community campaigns or actions on CVD risk prevention (e.g. stop smoking campaigns, fun-runs etc)?
• Does the practice use a CVD standardized risk assessment tool?
• Is the CVD risk assessment tool integrated with the patient medical record system (e.g. so that the CVD event risk score is entered directly in to the patient's medical record)?
• Is there in general a record in the electronic or paper based patient record that the CVD standardized risk assessment tool has been offered?
• Is CVD risk advice (e.g. about modifiable risk factors such as diet and exercise) integrated with the patient medical record system?
• Do you offer regularly two or many consultations to provide advice on patient's life style?
• Does the practice have an up-to-date directory of prevention activities/organizations available locally (e.g. gyms, walking group, weight-watchers etc)?
• Did your practice participate in a project concerning cardiovascular risk management the last 2 years (apart from those mentioned above)?

For multivariable prediction, a series of linear models were estimated to assess the effect of variables at practice and patient-level on HRQoL; explanatory variables at country level were not examined. Because of hierarchical data structure, multilevel analysis was performed to take into account the dependence between patient outcomes (level 1) within primary care practices (level 2) and countries (level 3). The multilevel linear analysis started with a three-level null (empty) model with no predictor variables in the fixed part and only the intercepts in the random part of the model (M1). This model can be used as reference for comparing the size of contextual (practice or country) variations in EQ-5D in subsequent models. Next, two practice-level performance characteristics (practice CVD care and practice quality management) were included as fixed effects (M2). Finally, we added patient-level variables in the fixed part of the third and fourth model (M3 and M4). However, in contrast to M3 that included only relevant patient characteristics on patient-level, M4 additionally contained variables measured at patient-level but reflecting aspects of care delivery.

We first present descriptive statistics for practice-level and patient-level characteristics in the entire study sample and then subgroup analyses for single-handed and group practices. Continuous data are summarized by using means with standard deviations (SD). Categorical data are presented as frequency counts and percentages. We then report on fixed-part results of the final 3-level linear model (M4) followed by the random-part results of all four models (M1–M4). Variance partition coefficients in each level were calculated using the restricted maximum likelihood (REML) method; the corresponding intraclass correlation coefficient (ICC) at the practice and country level [35] is provided. Finally, the proportion of variance explained (EV) at each level [36] is presented for model M2–M4.

Only patients with complete data on all explanatory variables were considered in the final model and included in the analysis. The characteristics of these patients were compared with those of the patients who had to be excluded because of non-responding to the EQ-5D items or lack of information on explanatory variables.

Because this was an exploratory analysis, the significance level was set to 5% (two-sided) and no adjustment for multiple testing was performed. All descriptive analyses were carried out by using IBM SPSS Statistics version 19 (SPSS Inc., Chicago, IL, USA). The multilevel analysis was conducted by using MLwiN 2.24 [37].

## Results

### Sample characteristics

For 3505 patients with CHD in 228 practices we were able to match data from survey instruments for patients and practices (Figure 1). Of the 228 participating practices, 30.7% were located in towns with more than 100,000 inhabitants and employed approximately two GP full time equivalents (FTE). Mean practice quality scores were 8.2 for CVD-care and 9.0 for quality management, respectively (Table 4).

**Table 4:** Sample description (included patients).

	Total sample		Single practice		Group practice		p-value
**Practice-level**							
Included practices	228		113		102		
FTE GP (SD)	2.04 (1.70)		1.00 (0.04)		3.20 (1.89)		
Practice town (>100.000 inhabitants) (%)	69 (30.7)		27 (23.9)		39 (39.4)		.0150^b^
CVD-care score (SD)	8.24 (4.72)		6.80 (3.97)		9.79 (5.05)		<.0001^a^
Quality-management score (SD)	9.00 (3.87)		8.05 (3.40)		10.42 (3.93)		<.0001^a^
**Patient-level**							
Included patients	2656		1341		1128		
EQ-5D score (SD)	0.73 (0.22)		0.73 (0.22)		0.73 (0.22)		.6838^a^
*Patient characteristics*							
Age (SD)	67.5 (10.1)		67.9 (10.2)		67.1 (9.9)		.0543^a^
Gender (female) (%)	809 (30.5)		405 (30.2)		360 (31.9)		.3590^b^
Marital status (single) (%)	623 (23.5)		305 (22.7)		279 (24.7)		.2465^b^
Years of education (< = 9 years) (%)	841 (31.7)		491 (36.6)		289 (25.6)		<.0001^b^
Number of other conditions (SD)	3.49 (1.79)		3.55 (1.86)		3.36 (1.70)		.0100^a^
Body mass index (> = 30) (%)	610 (23.0)		270 (20.1)		291 (25.8)		.0008^b^
*Care delivery*							
Being patient in practice							.0098^b^
– up to 2 years (%)	119 (4.5)		46 (3.4)		63 (5.6)		
– 3–7 years (%)	346 (13.0)		187 (13.9)		130 (11.5)		
– more than 7 years (%)	2191 (82.5)		1108 (82.6)		935 (82.9)		
Practice attendance within 12 months							<.0001^b^
– up to 3 times (%)	737 (27.7)		316 (23.6)		381 (33.8)		
– 4–7 times (%)	1189 (44.8)		589 (43.9)		489 (43.4)		
– more than 7 times (%)	730 (27.5)		436 (32.5)		258 (22.9)		
Referral to exercise program (yes) (%)	1273 (47.9)		660 (49.2)		529 (46.9)		.2505^b^
Medication adherence (SD)	3.49 (0.81)		3.47 (0.85)		3.52 (0.78)		.1702^a^
Patient satisfaction (EUROPEP)							
– clinical behavior (SD)	4.44 (0.66)		4.44 (0.63)		4.44 (0.70)		.8626^a^
– organization of care (SD)	4.39 (0.68)		4.43 (0.63)		4.33 (0.74)		.0003^a^

a = T-Test (Single vs. Group);

b = Chi^2^ (Single vs. Group); CVD: Cardiovascular disease.

After excluding patients with missing information, our cohort for further analysis consisted of 2656 patients (75.8% of 3505). Table 4 shows the socio-demographic characteristics as well as characteristics of care delivery of the included patients. Compared to those included, the patients excluded from further analysis were of similar mean age (68.5 years), proportion of female (36%), proportion of patients with BMI of 30 or above (23%), proportion of patients with nine or less years of education (30%), and single marital status (27%). However, those excluded had slightly fewer other conditions and lower medication adherence, and were less likely to be referred to an exercise program (41% vs. 48%). The HRQoL of the patients without missing values was similar to the HRQoL of the patients excluded from further analysis: The mean EQ-5D was 0.73 (0.22) and 0.72 (0.23), respectively.

Significant differences between single and group practices were not found for HRQoL but for some explanatory variables (Table 4). At practice-level, group practices scored higher on both practice quality dimensions and were more often located in larger towns, compared with single-handed practices. At the patient-level, the percentage of patients with BMI greater or equal to 30 was higher at group practices than in single-handed practices (26% vs. 20%). Similarly, the proportion of patients with more than nine years of education was higher in group practices compared to single-handed practices (74% vs. 63%). In contrast, patients in single-handed practices were more likely to attend the practice more than seven times within one year and were also slightly more often referred to an exercise program.

### Multivariable associations with HRQoL

The 3-level linear regression analysis was based on 2656 patients (level 1) nested within 228 practices (level 2) and 8 countries (level 3). There were up to 36 patients within each practice and up to 34 practices within each country. The multilevel regression analysis showed that the HRQoL as measured by the EQ-5D score was associated with several variables, especially at the patient-level. In contrast, none of the variables at practice-level were significantly associated with the EQ-5D score (Table 5).

**Table 5:** Fixed part results of the random intercept model with overall EQ-5D score as dependent variable (2656 patients within 228 GPs within 8 countries), including all variables on patient and practice-level.

	Total sample*			Single practices**			Group practices***		
	coeff.	(SE)	p-value	coeff.	(SE)	p-value	coeff.	(SE)	p-value
Intercept	0.7047	(0.0467)	<.0001	0.8296	(0.0640)	<.0001	0.6009	(0.0667)	<.0001
**Practice-level**									
CVD-care score	−0.0017	(0.0017)	.3185	−0.0032	(0.0024)	.1786	−0.0011	(0.0025)	.6517
Quality-management score	0.0021	(0.0018)	.2231	0.0028	(0.0025)	.2759	0.0011	(0.0025)	.6697
**Patient-level**									
*Patient characteristics*									
Age (5-years unit)	−0.0064	(0.0019)	.0008	−0.0101	(0.0027)	.0001	−0.0036	(0.0030)	.2374
Gender (female)	−0.0543	(0.0083)	<.0001	−0.0439	(0.0118)	.0002	−0.0662	(0.0125)	<.0001
Marital status (single)	−0.0142	(0.0088)	.1074	−0.0294	(0.0127)	.0205	−0.0037	(0.0133)	.7808
Years of education (< = 9 years in school)	−0.0381	(0.0083)	<.0001	−0.0471	(0.0112)	<.0001	−0.0296	(0.0137)	.0304
Number of other conditions	−0.0340	(0.0022)	<.0001	−0.0367	(0.0030)	<.0001	−0.0319	(0.0035)	<.0001
Body mass index (> = 30)	−0.0211	(0.0086)	.0145	−0.0127	(0.0127)	.3165	−0.0279	(0.0130)	.0314
*Care delivery*									
Being patient in practice			.0473			.0320			.5838
– up to 2 years	−0.0214	(0.0176)		−0.0394	(0.0280)		−0.0256	(0.0248)	
– 3–7 years	−0.0250	(0.0110)		−0.0352	(0.0150)		−0.0041	(0.0181)	
– more than 7 years	*Reference*			*Reference*			*Reference*		
Practice attendance within 12 months			<.0001			<.0001			<.0001
– up to 3 times	0.1210	(0.0109)		0.0940	(0.0156)		0.1566	(0.0165)	
– 4–7 times	0.0801	(0.0091)		0.0731	(0.0126)		0.0910	(0.0146)	
– more than 7 times	*Reference*			*Reference*			*Reference*		
Referral to exercise program (yes)	0.0240	(0.0075)	.0013	0.0135	(0.0107)	.2047	0.0394	(0.0115)	.0006
Medication adherence	0.0195	(0.0045)	<.0001	0.0179	(0.0062)	.0039	0.0180	(0.0072)	.0117
Patient satisfaction (EUROPEP)									
– clinical behavior	0.0282	(0.0076)	.0002	0.0335	(0.0114)	.0034	0.0324	(0.0111)	.0035
– organization of care	−0.0032	(0.0075)	.6737	−0.0142	(0.0116)	.2194	0.0007	(0.0109)	.9523

coeff.: regression coefficient, SE: standard error, CVD: Cardiovascular disease.

* 2656 patients, 228 practices, 8 countries.

** 1341 patients, 113 practices, 8 countries.

*** 1128 patients, 102 practices, 8 countries.

At the patient-level all socio-demographic characteristics were significantly associated with HRQoL except for marital status. In terms of practical impact, the regression coefficient as result of the multilevel analysis has to be interpreted carefully. In general, the value of the regression coefficient indicates a change per unit (continuous variables) or a change in contrast to a reference category (categorical variables). For example, the continuous variable “age” is in our analysis divided in 5 years units. This means, that in steps of 5 years (one unit) the EQ-5D score decreases (negative sign) by 0.0064 (regression coefficient). For the categorical variable “gender” the regression coefficient indicates a change of −0.0543 on the EQ-5D score for “female” in contrast to “male” patients (reference category). Additionally, a higher number of other conditions (regression coefficient: −0.0340; 11 units), and BMI of 30 or above (regression coefficient: −0.0211) were associated with lower HRQoL.

The relationship between the variables of care delivery and HRQoL was almost as expected. Shorter duration of being patient in practice had a negative effect on HRQoL (regression coefficients: −0.0214 for up to 2 years and −0.0250 for 3–7 years compared with more than 7 years, respectively). Patients, who were attending their practices less often, had a higher EQ-5D score compared to patients who reported to attend their practice more than 7 times within one year. All the other factors concerning care delivery, except for the organizational subscale score of the EUROPEP score (regression coefficient: −0.0032, p = 0.6737), were positively associated with HRQoL. Full information on fixed-part results from all four models can be found in Tables S1,S2,S3.

**Figure 2:**
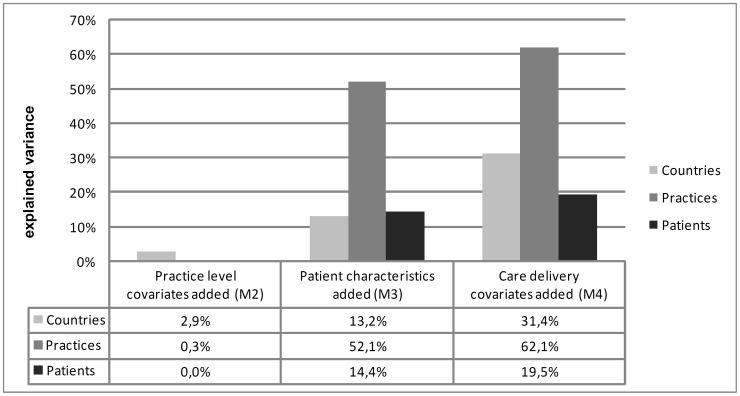
Proportion of variance in overall EQ-5D score explained at each level.

With respect to the random part of the results the null (empty) model (M1) showed a total variation in EQ-5D of 0.0405. The ICC for the country level was 0.08 and 0.06 for the practice-level. No reduction in EQ-5D variance could be observed after controlling for practice-level characteristics (M2). In contrast, including patient's socio-demographic variables (M3) resulted in a considerable reduction of the observed variance. Further reduction of the variance in EQ-5D was reached by adding variables of care delivery (M4) (Table 6). The final model explained the variance at the country level to 31.4%, at the practice-level to 62.1% and at the patient-level to 19.6% (Figure 2).

**Table 6:** Random part of all three random intercept models with overall EQ-5D score as dependent variable (2656 individuals within 228 GPs within 8 countries).

	All practices			Single-handed practice			Group practice		
	VC	(SE)	EV	VC	(SE)	EV	VC	(SE)	EV
**Model 1: Null model**									
Countries	0.0038	(0.0020)		0.0034	(0.0020)		0.0047	(0.0028)	
Practices	0.0029	(0.0006)		0.0036	(0.0010)		0.0020	(0.0009)	
Patients (plus random)	0.0405	(0.0012)		0.0411	(0.0017)		0.0428	(0.0019)	
**Model 2: Practice-level covariates added**									
Countries	0.0037	(0.0020)	2.90%	0.0032	(0.0019)	5.62%	0.0044	(0.0026)	6.01%
Practices	0.0029	(0.0006)	0.34%	0.0034	(0.0010)	3.93%	0.0021	(0.0009)	NA
Patients (plus random)	0.0405	(0.0012)	0.00%	0.0411	(0.0017)	NA	0.0428	(0.0019)	0.05%
**Model 3: Patient characteristics added**									
Countries	0.0033	(0.0017)	13.19%	0.0023	(0.0013)	32.25%	0.0039	(0.0223)	16.74%
Practices	0.0014	(0.0004)	52.07%	0.0012	(0.0006)	67.42%	0.0014	(0.0007)	27.92%
Patients (plus random)	0.0347	(0.0010)	14.36%	0.0342	(0.0014)	16.70%	0.0369	(0.0016)	13.83%
**Model 4: Care delivery added**									
Countries	0.0026	(0.0014)	31.40%	0.0016	(0.0010)	52.37%	0.0018	(0.0011)	61.37%
Practices	0.0011	(0.0004)	62.07%	0.0011	(0.0005)	69.38%	0.0006	(0.0005)	69.04%
Patients (plus random)	0.0326	(0.0009)	19.55%	0.0327	(0.0013)	20.35%	0.0335	(0.0015)	21.66%

VC: Variance component, SE: standard error, EV: explained variance, NA: not available.

### Single-handed and group practices

The results of the final model were similar when single-handed practices and group practices were considered separately. However, for patients in single-handed practices ‘single marital status’ had a statistically significant negative effect on the EQ-5D score, whereas a negative but statistically non-significant association was observed between BMI and HRQoL and ‘referral to exercise program’ and HRQoL. In group practices, a non-significant association with HRQoL was observed for patient's age and the time of being patient in practice.

The random-part analysis for group and single-handed practices (Table 6) revealed a greater importance of patient characteristics in single-handed practice compared to group practices. Including of ‘patient characteristics’ in the multilevel model resulted at the practice-level in an increase explained variance to 67%. However, in group practices the explained variance increased only to 28%. In contrast, including variables of ‘care delivery’ explained more variance in group practices (28% to 69%) compared to single-handed practices (67% to 69%).

## Discussion

The aim of this observational study was to identify factors which are associated with HRQoL in usual primary care at practice and patient-level. Relating to the practice-level, neither the score of the dimension ‘quality-management’ nor the score ‘practice CVD-care’ were associated with HRQoL in our analysis. However, the level of these scores was quite low. On average, only 60% of the quality-management items (mean 9.0; range 0–15) and 48% of CVD-care items (mean 8.2; range 0–17) respectively, were implemented. Moreover, the standard deviations indicated that there were large differences between practices. These differences could also be seen when comparing single-handed and group practices. Group practices scored significant higher on quality indicators compared to single-handed practices. However, the EQ-5D scores in both groups were similar. The subgroup analysis revealed that the measured aspects of practice organization had no significant impact on HRQoL. This lack of association between practice characteristics and HRQoL was not expected, as aspects of good practice organization have been shown to improve quality of care in previous studies [38]–[40]. On the other hand, our findings do not exclude a positive or negative impact of practice organization on HRQoL over time due to the cross-sectional study design.

At patient-level, lower education, female gender and a higher number of other conditions, had the strongest negative impact on HRQoL. Whereas the negative impact of lower education, female gender and individual conditions (e.g. hypertension) is well known [22], [41], [42], the association between the number of conditions and HRQoL has not been explicitly demonstrated in patients with CHD. To date, the correlation between HRQoL and the number of conditions was mainly known for other diseases, like diabetes [43].

The strongest positive association with HRQoL was found in our study for good medication adherence (Morisky) and a higher score in the EUROPEP dimension ‘clinical behavior’ (indicating a good patient-doctor relationship). As good medication adherence is (among other aspects) influenced by interaction between patients and treating physicians [44], [45], both variables underline the importance of patient-doctor relationship on HRQoL. Previous research has shown that patient-physician relationship can be linked to improved health outcomes [46].

Particularly, good communication between doctors and patients could lead to better physical health by identifying the diagnosis, finding an appropriate treatment plan or strengthening self-management in chronic care. Additionally, positive effects on psychosocial outcomes can be a result of patient-centered communication from which patients feel recognized, validated, worthy, reassured, and comforted [46]. In secondary prevention and chronic care, a good patient-physician relationship is the basis of a multifaceted regimen, which involves long-term management of risk factors, support of medication adherence and lifestyle interventions, like obesity or physical activity counseling [47]–[50]. From this point of view, potential for improvements in HRQoL particularly exist in strengthening the patient-doctor relationship.

### Strengths and limitations

The EPA cardio study is one of the largest international studies on the management of cardiovascular prevention in European primary care. We used multilevel modeling to account for the hierarchical data structure and to identify predictors of HRQoL adjusting for all other variables. Hierarchical models combine information across units to produce accurate and well calibrated prediction of outcomes. This analytic approach has been found to be very relevant in health services research as patients' data were similarly clustered at more than one level. We used validated measures and collected morbidity data from medical records in contrast to self-reported morbidity indicators that could lead to misclassifications.

Nevertheless, in some countries it was difficult to enroll 36 practices as intended. In the multivariable analyses, the total number of cases decreased due to missing data, as we conducted a complete case analysis. The EQ-5D instrument showed a ceiling effect with 30% of people scoring the highest value. As also reported in other studies, EQ-5D may be less sensitive to describe mild severity health levels. However, the EQ-5D instrument is reported to have a better discrimination capacity for socio-demographic and morbidity indicators that were focused in our study. Because of the observational design of our study, the correlations found cannot be used to attest causal associations.

### Conclusion

The results of our study may suggest that the patient-physician relationship rather than the organization of CVD care and aspects of quality management is an important predictor of health status in usual primary care. This is also reflected in the finding, that no differences between single-handed and group practices concerning HRQoL exist. For further development, the results may imply that interventions to improve HRQoL require a strong patient-centered approach. That the majority of patients, as shown by our results, have a long-term relationship to their primary care practice, is a good basis for further developments in this field.

## Supporting Information

Table S1 Fixed part results of the random intercept models fitted to the total sample.
(DOCX)

Table S2 Fixed part results of the random intercept models fitted to subsample of single- handed practices.
(DOCX)

Table S3 Fixed part results of the random intercept models fitted to subsample of group practices.
(DOCX)
