# Supplementary material for: Secondary Prevention in Patients with Coronary Heart Diseases: What Factors Are Associated with Health Status in Usual Primary Care?
Source: PLoS One. 2012 Dec 26;7(12):e51726. doi: 10.1371/journal.pone.0051726 (PMC3530503; doi:10.1371/journal.pone.0051726)
Supplement: Table S1 — Fixed part results of the random intercept models fitted to the total sample. (DOCX) [file pone.0051726.s001.docx]

Table S1: Fixed part results of the random intercept models fitted to the total sample

|  | **null model** | | | **practice scores** (added) | | | **patient attributes** (added) | | | **care delivery** (added) | | |
| --- | --- | --- | --- | --- | --- | --- | --- | --- | --- | --- | --- | --- |
|  | coeff. | (SE) | p-value | coeff. | (SE) | p-value | coeff. | (SE) | p-value | coeff. | (SE) | p-value |
| Intercept | 0.7259 | (0.0225) | <.0001 | 0.7387 | (0.0300) | <.0001 | 1.0106 | (0.0373) | <.0001 | 0.7047 | (0.0467) | <.0001 |
| **Practice level** |  |  |  |  |  |  |  |  |  |  |  |  |
| CVD-care score |  |  |  | -0.0044 | (0.0022) | .0438 | -0.0027 | (0.0018) | .1419 | -0.0017 | (0.0017) | .3185 |
| Quality-management score |  |  |  | 0.0024 | (0.0022) | .2803 | 0.0025 | (0.0019) | .1875 | 0.0021 | (0.0018) | .2231 |
| **Patient level** |  |  |  |  |  |  |  |  |  |  |  |  |
| *Chararacteristics* |  |  |  |  |  |  |  |  |  |  |  |  |
| Age (5-years unit) |  |  |  |  |  |  | -0.0083 | (0.0019) | <.0001 | -0.0064 | (0.0019) | .0008 |
| Gender (female) |  |  |  |  |  |  | -0.0679 | (0.0084) | <.0001 | -0.0543 | (0.0083) | <.0001 |
| Marital status (single) |  |  |  |  |  |  | -0.0209 | (0.0090) | .0205 | -0.0142 | (0.0088) | .1074 |
| Years of education (<= 9 years in school) |  |  |  |  |  |  | -0.0420 | (0.0086) | <.0001 | -0.0381 | (0.0083) | <.0001 |
| Number of other conditions |  |  |  |  |  |  | -0.0377 | (0.0022) | <.0001 | -0.0340 | (0.0022) | <.0001 |
| BMI (>= 30) |  |  |  |  |  |  | -0.0294 | (0.0089) | .0009 | -0.0211 | (0.0086) | .0145 |
| *Care delivery* |  |  |  |  |  |  |  |  |  |  |  |  |
| Being patient in practice |  |  |  |  |  |  |  |  |  |  |  | .0473 |
| − up to 2 years |  |  |  |  |  |  |  |  |  | -0.0214 | (0.0176) |  |
| − 3-7 years |  |  |  |  |  |  |  |  |  | -0.0250 | (0.0110) |  |
| − more than 7 years |  |  |  |  |  |  |  |  |  | *Reference* | | |
| Practice attendance within 12 months |  |  |  |  |  |  |  |  |  |  |  | <.0001 |
| **−** up to 3 times |  |  |  |  |  |  |  |  |  | 0.1210 | (0.0109) |  |
| − 4-7 times |  |  |  |  |  |  |  |  |  | 0.0801 | (0.0091) |  |
| − more than 7 times |  |  |  |  |  |  |  |  |  | *Reference* | | |
| Evaluation of practice care |  |  |  |  |  |  |  |  |  |  |  |  |
| − clinical behavior |  |  |  |  |  |  |  |  |  | 0.0282 | (0.0076) | .0002 |
| − organization of care |  |  |  |  |  |  |  |  |  | -0.0032 | (0.0075) | .6737 |
| Referral to excercise program (yes) |  |  |  |  |  |  |  |  |  | 0.0240 | (0.0075) | .0013 |
| Medication adherence |  |  |  |  |  |  |  |  |  | 0.0195 | (0.0045) | <.0001 |
| coeff.: regression coefficient, SE: standard error, CVD: Cardiovascular disease, BMI: body mass index | | | | | |  |  |  |  |  |  |  |
