# Supplementary material for: Secondary Prevention in Patients with Coronary Heart Diseases: What Factors Are Associated with Health Status in Usual Primary Care?
Source: PLoS One. 2012 Dec 26;7(12):e51726. doi: 10.1371/journal.pone.0051726 (PMC3530503; doi:10.1371/journal.pone.0051726)
Supplement: Table S2 — Fixed part results of the random intercept models fitted to subsample of single- handed practices. (DOCX) [file pone.0051726.s002.docx]

Table S2: Fixed part results of the random intercept models fitted to subsample of single handed practices

|  | **null model** | | | **practice scores** (added) | | | **patient attributes** (added) | | | **care delivery** (added) | | |
| --- | --- | --- | --- | --- | --- | --- | --- | --- | --- | --- | --- | --- |
|  | coeff. | (SE) | p-value | coeff. | (SE) | p-value | coeff. | (SE) | p-value | coeff. | (SE) | p-value |
| Intercept | 0.7288 | (0.0226) | <.0001 | 0.7516 | (0.0348) | <.0001 | 1.0922 | (0.0453) | <.0001 | 0.8296 | (0.0640) | <.0001 |
| **Practice level** |  |  |  |  |  |  |  |  |  |  |  |  |
| CVD-care score |  |  |  | -0.0065 | (0.0032) | .0423 | -0.0039 | (0.0025) | .1225 | -0.0032 | (0.0024) | .1786 |
| Quality-management score |  |  |  | 0.0030 | (0.0034) | .3778 | 0.0029 | (0.0026) | .2643 | 0.0028 | (0.0025) | .2759 |
| **Patient level** |  |  |  |  |  |  |  |  |  |  |  |  |
| *Chararacteristics* |  |  |  |  |  |  |  |  |  |  |  |  |
| Age (5-years unit) |  |  |  |  |  |  | -0.0130 | (0.0027) | <.0001 | -0.0101 | (0.0027) | .0001 |
| Gender (female) |  |  |  |  |  |  | -0.0520 | (0.0119) | <.0001 | -0.0439 | (0.0118) | .0002 |
| Marital status (single) |  |  |  |  |  |  | -0.0366 | (0.0129) | .0046 | -0.0294 | (0.0127) | .0205 |
| Years of education (<= 9 years in school) |  |  |  |  |  |  | -0.0512 | (0.0114) | <.0001 | -0.0471 | (0.0112) | <.0001 |
| Number of other conditions |  |  |  |  |  |  | -0.0402 | (0.0030) | <.0001 | -0.0367 | (0.0030) | <.0001 |
| BMI (>= 30) |  |  |  |  |  |  | -0.0169 | (0.0129) | .1896 | -0.0127 | (0.0127) | .3165 |
| *Care delivery* |  |  |  |  |  |  |  |  |  |  |  |  |
| Being patient in practice |  |  |  |  |  |  |  |  |  |  |  | .0320 |
| − up to 2 years |  |  |  |  |  |  |  |  |  | -0.0394 | (0.0280) |  |
| − 3-7 years |  |  |  |  |  |  |  |  |  | -0.0352 | (0.0150) |  |
| − more than 7 years |  |  |  |  |  |  |  |  |  | *Reference* | | |
| Practice attendance within 12 months |  |  |  |  |  |  |  |  |  |  |  | <.0001 |
| **−** up to 3 times |  |  |  |  |  |  |  |  |  | 0.0940 | (0.0156) |  |
| − 4-7 times |  |  |  |  |  |  |  |  |  | 0.0731 | (0.0126) |  |
| − more than 7 times |  |  |  |  |  |  |  |  |  | *Reference* | | |
| Evaluation of practice care |  |  |  |  |  |  |  |  |  |  |  |  |
| − clinical behavior |  |  |  |  |  |  |  |  |  | 0.0335 | (0.0114) | .0034 |
| − organization of care |  |  |  |  |  |  |  |  |  | -0.0142 | (0.0116) | .2194 |
| Referral to excercise program (yes) |  |  |  |  |  |  |  |  |  | 0.0135 | (0.0107) | .2047 |
| Medication adherence |  |  |  |  |  |  |  |  |  | 0.0179 | (0.0062) | .0039 |
| coeff.: regression coefficient, SE: standard error, CVD: Cardiovascular disease, BMI: body mass index | | | | | |  |  |  |  |  |  |  |
