# Supplementary material for: Secondary Prevention in Patients with Coronary Heart Diseases: What Factors Are Associated with Health Status in Usual Primary Care?
Source: PLoS One. 2012 Dec 26;7(12):e51726. doi: 10.1371/journal.pone.0051726 (PMC3530503; doi:10.1371/journal.pone.0051726)
Supplement: Table S3 — Fixed part results of the random intercept models fitted to subsample of group practices. (DOCX) [file pone.0051726.s003.docx]

Table S3: Fixed part results of the random intercept models fitted to subsample of group practices

|  | **null model** | | | **practice scores** (added) | | | **patient attributes** (added) | | | **care delivery** (added) | | |
| --- | --- | --- | --- | --- | --- | --- | --- | --- | --- | --- | --- | --- |
|  | coeff. | (SE) | p-value | coeff. | (SE) | p-value | coeff. | (SE) | p-value | coeff. | (SE) | p-value |
| Intercept | 0.7264 | (0.0272) | <.0001 | 0.7439 | (0.0422) | <0,0001 | 0.9576 | (0.0573) | <.0001 | 0.6009 | (0.0667) | <.0001 |
| **Practice level** |  |  |  |  |  |  |  |  |  |  |  |  |
| CVD-care score |  |  |  | -0.0032 | (0.0033) | .3415 | -0.0013 | (0.0030) | .6533 | -0.0011 | (0.0025) | .6517 |
| Quality-management score |  |  |  | 0.0009 | (0.0033) | .7853 | 0.0013 | (0.0030) | .6727 | 0.0011 | (0.0025) | .6697 |
| **Patient level** |  |  |  |  |  |  |  |  |  |  |  |  |
| *Chararacteristics* |  |  |  |  |  |  |  |  |  |  |  |  |
| Age (5-years unit) |  |  |  |  |  |  | -0.0046 | (0.0031) | .1364 | -0.0036 | (0.0030) | .2374 |
| Gender (female) |  |  |  |  |  |  | -0.0910 | (0.0130) | <.0001 | -0.0662 | (0.0125) | <.0001 |
| Marital status (single) |  |  |  |  |  |  | -0.0094 | (0.0139) | .4976 | -0.0037 | (0.0133) | .7808 |
| Years of education (<= 9 years in school) |  |  |  |  |  |  | -0.0336 | (0.0145) | .0206 | -0.0296 | (0.0137) | .0304 |
| Number of other conditions |  |  |  |  |  |  | -0.0364 | (0.0035) | <.0001 | -0.0319 | (0.0035) | <.0001 |
| BMI (>= 30) |  |  |  |  |  |  | -0.0404 | (0.0136) | .0030 | -0.0279 | (0.0130) | .0314 |
| *Care delivery* |  |  |  |  |  |  |  |  |  |  |  |  |
| Being patient in practice |  |  |  |  |  |  |  |  |  |  |  | .5838 |
| − up to 2 years |  |  |  |  |  |  |  |  |  | -0.0256 | (0.0248) |  |
| − 3-7 years |  |  |  |  |  |  |  |  |  | -0.0041 | (0.0181) |  |
| − more than 7 years |  |  |  |  |  |  |  |  |  | *Reference* | | |
| Practice attendance within 12 months |  |  |  |  |  |  |  |  |  |  |  | <.0001 |
| **−** up to 3 times |  |  |  |  |  |  |  |  |  | 0.1566 | (0.0165) |  |
| − 4-7 times |  |  |  |  |  |  |  |  |  | 0.0910 | (0.0146) |  |
| − more than 7 times |  |  |  |  |  |  |  |  |  | *Reference* | | |
| Evaluation of practice care |  |  |  |  |  |  |  |  |  |  |  |  |
| − clinical behavior |  |  |  |  |  |  |  |  |  | 0.0324 | (0.0111) | .0035 |
| − organization of care |  |  |  |  |  |  |  |  |  | 0.0007 | (0.0109) | .9523 |
| Referral to excercise program (yes) |  |  |  |  |  |  |  |  |  | 0.0394 | (0.0115) | .0006 |
| Medication adherence |  |  |  |  |  |  |  |  |  | 0.0180 | (0.0072) | .0117 |
| coeff.: regression coefficient, SE: standard error, CVD: Cardiovascular disease, BMI: body mass index | | | | | |  |  |  |  |  |  |  |
